# Public health round-up

**DOI:** 10.2471/BLT.22.010622

**Published:** 2022-06-01

**Measles threat grows**

A nurse prepares a dose of a measles vaccine for a child at a temporary “Zwakala” vaccination site in Thembisa, South Africa. The “Zwakala” campaign is primarily designed to increase uptake of coronavirus disease 2019 (COVID-19) vaccinations in young people, but also offers routine childhood immunizations, many of which have been missed during the pandemic. According to a 27 April statement by the World Health Organization (WHO) and the United Nations Children’s Fund (UNICEF), the risk for large outbreaks of measles has significantly increased due to routine immunization being halted or delayed.

**Figure:**
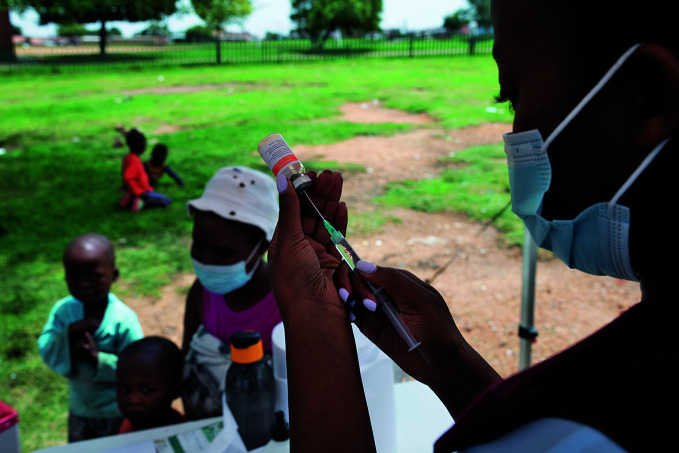
Daylin Paul/UNICEF

## New Ebola outbreak

The Ministry of Health of the Democratic Republic of the Congo declared an outbreak of Ebola virus disease in the country after a case was confirmed in Equateur province.

A 31-year-old male from Mbandaka began to have symptoms on 5 April and was treated at home before being admitted to two clinics in succession. He died on 21 April. The man’s sister-in-law was reported to be infected with Ebola virus on 25 April and died on 26 April.

As of 27 April, ring vaccination with the rVSV-ZEBOV Ebola vaccine had begun and a 20-bed Ebola treatment centre has been set up in Mbandaka, a city of approximately 1.2 million people. Disease surveillance and investigation of suspected cases was underway to detect any new infections, with WHO support that includes a team of six epidemiologists.

Because the man first infected was treated at home before being admitted to two health facilities and only isolated after the onset of bleeding, there is a significant risk of transmission.

This is the third Ebola outbreak in Equateur province and the country’s sixth since 2018.

https://bit.ly/3M9HTmY

## Measles outbreak concerns

The risk of significant outbreaks of measles has increased as a result of routine immunization being halted or delayed because of the COVID-19 pandemic. The risk has been further exacerbated by communities relaxing social distancing practices and other preventive measures implemented at the height of the pandemic, as noted by WHO and UNICEF in a 27 April statement.

Some 17 338 measles cases were reported worldwide in January and February 2022, compared with 9665 during the first two months of 2021. As of April 2022, the agencies were reporting 21 large and disruptive measles outbreaks around the world in the previous 12 months.

Most of the measles cases were reported in Africa and the Eastern Mediterranean Region, with the largest outbreaks occurring in Afghanistan, Ethiopia, Nigeria, Somalia and Yemen.

Because measles is highly contagious, cases tend to show up quickly when vaccination levels decline. There is a concern that that the outbreaks being reported may foreshadow outbreaks of other diseases in the coming months.

https://bit.ly/3P5XqGG

## COVID-19 technology licensing agreement

WHO’s COVID-19 Technology Access Pool and the Medicines Patent Pool finalized a licensing agreement with the United States National Institutes of Health for the development of several innovative therapeutics, early-stage vaccines and diagnostic tools for COVID-19.

Finalized on 12 May, the licences will allow manufacturers worldwide to make products ranging from the stabilized spike protein used in currently available COVID-19 vaccines, to early-stage vaccine candidates and diagnostics. The aim is to make such products accessible to all countries.

https://bit.ly/3FIAJnp

## Japanese encephalitis in Australia

In Australia, the government declared a Communicable Disease Incident of National Significance in response to its first, locally acquired Japanese encephalitis cases. The first case was reported on 3 March 2022 in Queensland. As of 28 April 2022, the Department of Health had reported that 37 people in four states had been infected, three of whom had died.

Serological evidence of exposure to the virus is periodically detected in animals in the Torres Strait Islands off the north coast of Queensland, but transmission on the mainland has not been previously established. This event, therefore, represents a significant change in the presence of the virus in Australia. The virus does not transmit between humans, and the risk of spread at the regional and global level is assessed to be low.

A flavivirus related to dengue, yellow fever and West Nile viruses, Japanese encephalitis virus is spread by mosquitoes. The vast majority of people infected are asymptomatic. However, 30% to 50% of those developing symptoms may suffer permanent neurologic or psychiatric harm and the case-fatality rate can be as high as 30%.

https://bit.ly/3ys2rn0

## Avian influenza firsts

The National Health Commission of China notified WHO of one confirmed case of human infection with an avian influenza A (H3N8) virus – the first reported case of this particular virus being transmitted to a human. As of 9 May, no further cases had been detected among close contacts.

An investigation of the event was launched. The limited epidemiologic and virologic information available suggests that the virus had not acquired the capacity to sustain transmission among humans. The risk of the disease spreading among humans was assessed to be low.

In related news, a human case of avian influenza A (H5N1) virus was reported in the United States of America. A man was infected after working at a commercial poultry facility in Colorado where the virus was confirmed to be present in the poultry.

https://bit.ly/3Pb1cOR

https://bit.ly/3FGoKa3

## Pandemic-related deaths

An estimated 14.9 million people died from causes directly or indirectly associated with the COVID-19 pandemic between 1 January 2020 and 31 December 2021. This is according to new excess mortality estimates published by WHO on 5 May. Most of the excess deaths (84%) were concentrated in South-East Asia, Europe, and the Americas, with middle-income countries accounting for 81%.

Excess mortality is calculated as the difference between the number of deaths that have occurred and the number that would be expected in the absence of the pandemic based on data from earlier years. Excess mortality includes deaths associated with COVID-19 directly (due to the disease) or indirectly (due to the pandemic’s impact on health systems and society).

https://bit.ly/3KQSGRQ

## WHO ambulances sent to Ukraine

WHO delivered 20 all-terrain ambulances to the Ministry of Health of Ukraine. The ambulances will be used to ensure medical evacuation.

WHO Director-General Tedros Adhanom Ghebreyesus handed over the keys to the ambulances to the Deputy Minister of Health Iryna Mykychak in the city of Lviv on 8 May. Dr Tedros was in Ukraine for 3 days of meetings with senior government leaders and to assess the current health needs in Ukraine.

WHO also delivered generators and blood refrigerators to hospitals and, as of 8 May, had delivered 393 metric tonnes of emergency and medical supplies and equipment.

https://bit.ly/3M6lirB

**Cover photo**

A man shows a vaccination card during an oral cholera vaccination campaign in Aldhale’ City, Yemen.

**Figure:**
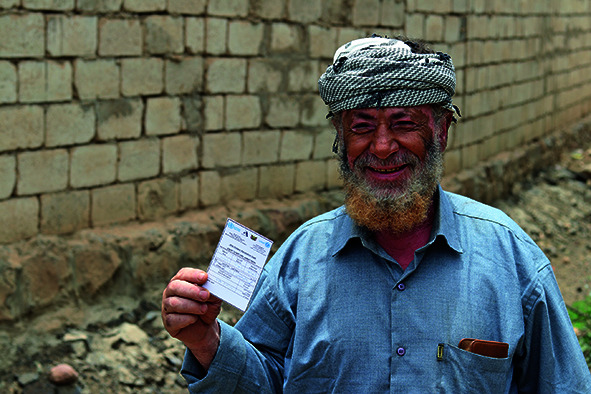
UNICEF/Mahmoud

## Preventing health-care-associated infections

An estimated 15 in 100 patients in acute-care hospitals in low- and middle-income countries will acquire at least one health-care-associated infection during their hospital stay. Roughly one in 10 affected patients will die as a result.

This is according to a new report on infection prevention and control published by WHO on 5 May which provides the first-ever global situation analysis of how infection prevention and control programmes are being implemented in countries around the world.

The report underlines the importance of good hand hygiene and other cost-effective infection prevention practices. Where such practices are followed, 70% of health-care-associated infections can be prevented.

The report also addresses the impact and cost-effectiveness of infection prevention and control programmes and the strategies and resources available to countries to improve them.

https://bit.ly/37ofyKO

## Aggressive breast-milk substitute marketing

A new WHO report titled *Scope and impact of digital marketing strategies for promoting breast-milk substitutes* sets out the wide range of digital marketing techniques being used by breast-milk substitute manufacturers to influence the decisions new families make on how to feed their babies and to gather information for targeted marketing purposes.

Released on 28 April, the report highlights the use of apps, virtual support groups or “baby-clubs”, paid social media influencers, promotions and competitions and advice forums or services, underlining the fact that formula milk companies buy or collect personal information to send personalized promotions to mothers.

The report summarizes findings of new research based on 4 million social media posts about infant feeding published between January and June 2021. These posts reached 2.47 billion people and generated more than 12 million likes, shares or comments.

https://bit.ly/3ygybex

**Looking ahead**

7–10 June, World Hepatitis Summit. https://bit.ly/3w5V4yF

30 June–1 July, High-level Meeting of the UN General Assembly on Global Road Safety. https://bit.ly/3MUNfT4

31 August–2 September, Health-enhancing physical activity Europe 2022 Conference. https://bit.ly/39xv07T

